# Liver zonation, revisited

**DOI:** 10.1002/hep.32408

**Published:** 2022-03-06

**Authors:** Jasmin Paris, Neil C. Henderson

**Affiliations:** ^1^ Centre for Inflammation Research The Queen’s Medical Research Institute Edinburgh BioQuarter University of Edinburgh Edinburgh UK; ^2^ MRC Human Genetics Unit Institute of Genetics and Cancer University of Edinburgh Edinburgh UK

## Abstract

The concept of hepatocyte functional zonation is well established, with differences in metabolism and xenobiotic processing determined by multiple factors including oxygen and nutrient levels across the hepatic lobule. However, recent advances in single‐cell genomics technologies, including single‐cell and nuclei RNA sequencing, and the rapidly evolving fields of spatial transcriptomic and proteomic profiling have greatly increased our understanding of liver zonation. Here we discuss how these transformative experimental strategies are being leveraged to dissect liver zonation at unprecedented resolution and how this new information should facilitate the emergence of novel precision medicine‐based therapies for patients with liver disease.

## Graphical abstract

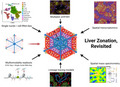

**Abbreviations**
FB: fibroblast
FISH: fluorescent in situ hybridization
GS: glutamine synthetase
Hamp: hepcidin antimicrobial peptide
HNF4α: hepatocyte nuclear factor 4 alpha
HSC: hepatic stellate cell
LGR: leucine‐rich repeat‐containing G protein‐coupled receptor
LSEC: liver sinusoidal endothelial cell
MARCO: macrophage receptor with collagenous structure
scRNA‐seq: single‐cell RNA sequencing
smFISH: single molecule FISH
snRA‐Seq: single‐nuclei RNA sequencing
VSMC: vascular smooth muscle cells

## INTRODUCTION

The concept of hepatocyte functional zonation has been known for some time, with differences in metabolism and xenobiotic processing determined by multiple factors including oxygen and nutrient levels across the hepatic lobule, bloodborne signaling molecules, and gradients of Wnt morphogens. However, before the advent of single‐cell genomics approaches, the inability to assess transcriptomic heterogeneity across cell subpopulations was a significant barrier to deepening our understanding of liver zonation. This review will focus on recent advances in the understanding of liver zonation, largely facilitated by a burgeoning array of single‐cell genomics technologies, such as single‐cell and single‐nuclei RNA sequencing, and the rapidly evolving field of spatial transcriptomic profiling.

## HISTORICAL PERSPECTIVES

Early descriptions of liver architecture were based on the distribution of the major hepatic blood vessels.^[^ ^1^, ^2^ ^]^ Rappaport extended this to encompass the microcirculatory and secretory capacity of the smallest structural units observed, thus formulating the concept of the liver acinus, with blood flowing in a portal to central direction and bile flowing the opposite way.^[^ ^3^, ^4^ ^]^ Rappaport was the first to describe the afferent, intermediate, and efferent zones of the liver (periportal Zone 1, midzone 2, and pericentral Zone 3), recognizing their differential nutrient and oxygen status, and damage susceptibility.^[^ ^5^, ^6^, ^7^ ^]^ Although Rappaport originally proposed a discrete periportal zone and continuous pericentral zone, subsequent staining for pericentral glutamine synthetase (GS) and periportal carbamoylphosphate in rat and human liver using the then‐novel technique of immunohistochemistry (IHC), demonstrated that the pericentral zone was in fact circular and discrete, whereas the periportal zone was reticular and continuous.^[^ ^8^, ^9^ ^]^

This work contributed to an expanding awareness of hepatocyte diversity, particularly relating to metabolic zonation across the lobule.^[^ ^10^, ^11^ ^]^ Further studies established that periportal (Zone 1) hepatocytes existing in an oxygen and nutrient‐rich environment carry out most of the liver’s metabolic functions, including beta oxidation, gluconeogenesis, urea and protein synthesis, and lipid metabolism. In contrast, pericentral (Zone 3) hepatocytes that exist in a low oxygen environment are characterized by glycolysis, xenobiotic biotransformation reactions, and glutamine synthesis.^[^ ^12^ ^]^

The first transcriptional study of liver zonation came from Braeuning et al., who carried out RNA microarray analysis of perivenous and periportal hepatocytes obtained by collagenase/digitonin perfusion of mouse liver.^[^ ^13^ ^]^ They identified 198 differentially expressed genes, 20 of which they validated by RT‐PCR. Differentially expressed genes included periportal genes involved in gluconeogenesis, fatty acid degradation, and amino acid degradation, contrasting with pericentral genes involved in glycolysis, cholesterol production, and xenobiotic metabolism. GS was expressed specifically in pericentral hepatocytes, consistent with the use of ammonia for glutamine synthesis in this zone. In contrast, at less stringent cutoffs, urea cycle enzymes were preferentially located in the periportal area, consistent with the use of ammonia for urea synthesis by these cells.

Further transcriptome analysis of liver zonation was carried out by McEnerney et al., who used laser capture microdissection to isolate periportal and pericentral zone hepatocytes before bulk RNA sequencing.^[^ ^14^ ^]^ Results demonstrated lipopolysaccharide (LPS)‐induced signaling in periportal hepatocytes and Wnt signaling and xenobiotic metabolism in pericentral hepatocytes.

## REGULATORS OF LIVER ZONATION

Unidirectional hepatic blood flow has long been known to regulate liver zonation,^[^ ^6^ ^]^ at least in part due to the presence of differing concentrations of oxygen present in periportal and perivenous blood.^[^ ^15^ ^]^

Further work has largely focused on Wnt signaling as a master regulator of liver zonation. In the presence of Wnt, cytoplasmic β‐catenin is released from its destruction complex and translocates to the nucleus to initiate gene transcription. Sekine et al. demonstrated that mice with liver specific β‐catenin deficiency lack pericentral GS staining and have significantly attenuated cytochrome P450 enzyme activity, indicating changes in metabolic zonation.^[^ ^16^ ^]^

Subsequent work identified a key role for Wnt in establishing liver zonation.^[^ ^17^, ^18^ ^]^ Benhamouche et al. demonstrated the complementary localization of activated β‐catenin in pericentral hepatocytes and the negative regulator Apc in periportal hepatocytes and demonstrated that Wnt/β‐catenin signaling inversely controls the pericentral and periportal genetic programs. They also showed that periportal urea cycle genes and pericentral glutamine synthesis genes are critical targets of β‐catenin and proposed that defective ammonia metabolism is responsible for the death of mice with liver *Apc* deficiency.^[^ ^17^ ^]^

Complementary work by Hailfinger et al. proposed a model for liver zonation based on differentially expressed genes observed in mice with experimentally induced liver tumors harboring mutations in either *β‐catenin* or *Ha Ras*.^[^ ^18^ ^]^ Comparing gene expression in these tumors with those observed in different liver zones of healthy mice, they proposed that β‐catenin produced by central vein endothelial cells regulates pericentral gene expression, whereas periportal gene expression is determined by Ras signaling, induced by bloodborne molecules arriving through the portal vein. Braeuning et al. confirmed the effect of serum components in maintaining periportal gene expression and also suggested that this was mediated by the Ras/MAP kinase extracellular pathway.^[^ ^19^ ^]^

The Rspondin family of Wnt agonists is known to promote Wnt signaling, because binding to their LGR receptors prevents membrane clearance of Wnt receptors. Consistent with the zonation of Wnt signaling, *LGR5* gene expression was demonstrated exclusively in pericentral hepatocytes.^[^ ^13^ ^]^ Subsequently, Rspo3 produced by central vein endothelial cells was shown to have a critical role in metabolic liver zonation,^[^ ^20^ ^]^ and ectopic expression of *Rspo1*, whose function is reported to be analogous to Rspo3 in terms of activating canonical β‐catenin signaling, resulted in expression of pericentral markers.^[^ ^20^ ^]^ Similar findings by Planas‐Paz et al. identified the RSPO–LGR4/5–ZNRF3/RNF43 module as a master regulator of Wnt/β‐catenin‐mediated metabolic liver zonation.^[^ ^21^ ^]^

In addition to Wnt morphogens, other factors reported to contribute to the maintenance of hepatic zonation include DICER^[^ ^22^ ^]^ and HNF4α.^[^ ^23^ ^]^ Specifically, Sekine et al. demonstrated that Dicer, an endoribonuclease III type enzyme required for microRNA biogenesis, plays an essential role in the establishment of proper liver zonation. Hepatocyte‐specific *Dicer* knockout mice showed impaired localization of periportal enzymes similar to those seen in *β‐catenin* knockout mice, although no direct relationship between *Dicer* and *β‐catenin* expression was identified.^[^ ^22^ ^]^ Stanulovic et al. showed that deficiency of the transcription factor *Hnf4α* results in ectopic expression of *GS* and other pericentral genes in the periportal zone.^[^ ^23^ ^]^

## ZONATION BY CELL TYPE

**FIGURE 1:**
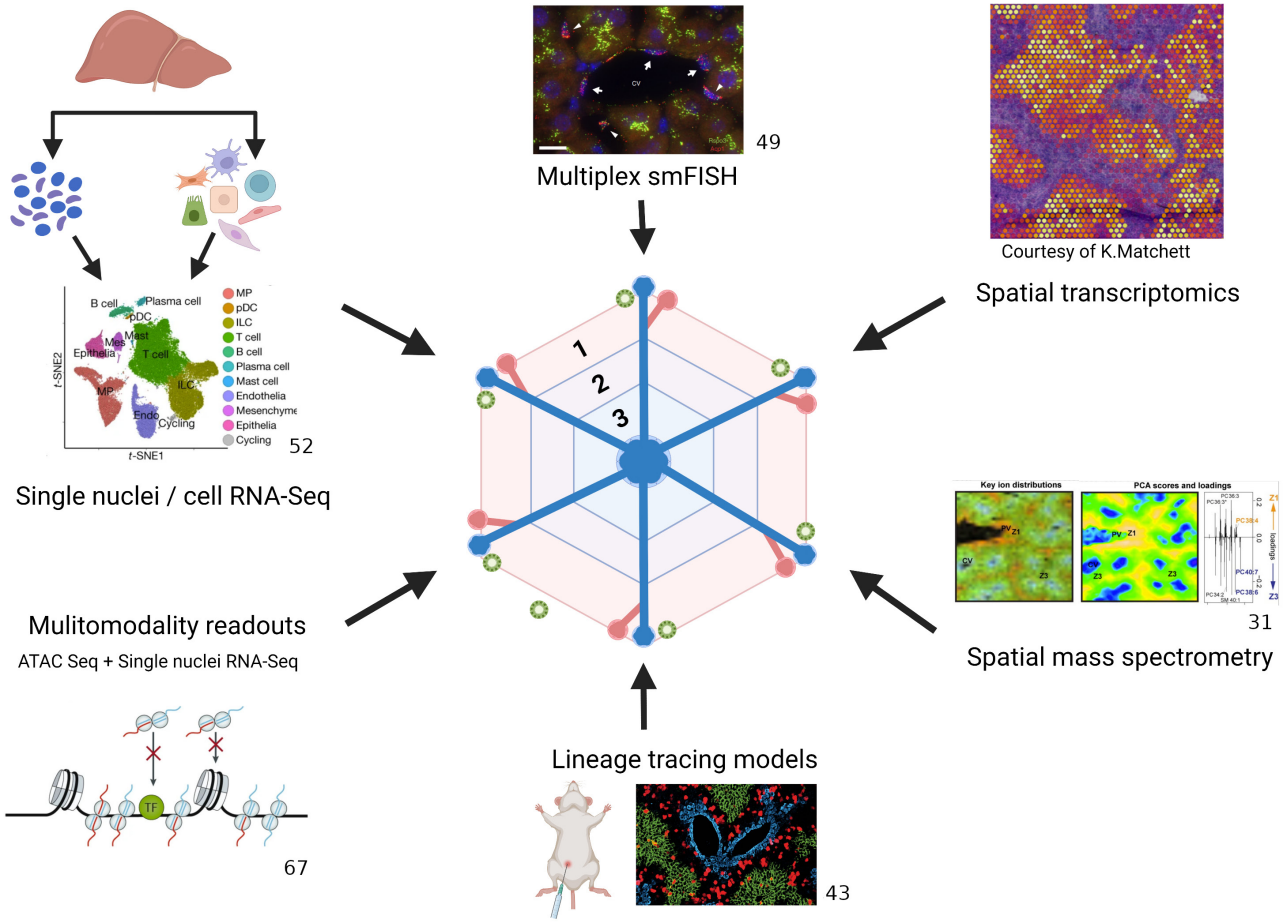
Technologies driving increased understanding of liver zonation. Transformative experimental strategies are being leveraged to dissect liver zonation at unprecedented resolution, including snRNA‐Seq and scRNA‐Seq,^[^ ^52^ ^]^ multiplex smFISH,^[^ ^49^ ^]^ spatial transcriptomics, spatial mass spectrometry,^[^ ^31^ ^]^ novel lineage tracing mouse models,^[^ ^43^ ^]^ and multimodality readouts such as the combination of ATAC‐Seq^[^ ^67^ ^]^ with snRNA‐Seq. Created with BioRender.com

Over the last few years, single‐cell RNA sequencing (scRNA‐Seq) and single‐nuclei RNA sequencing (snRNA‐Seq) technologies have revealed remarkable cellular heterogeneity within tissues, including the liver. In combination with the rapid development of novel spatial techniques, including multiplex single molecule FISH (smFISH) and spatial transcriptomics (Figure 1), these technologies have driven a step change in our understanding of liver zonation across a variety of cell types (Table 1), which will be discussed below. Methods for the computational analysis and integration of single‐cell transcriptomic and multiomic data sets have recently been reviewed elsewhere.^[^ ^24^, ^25^ ^]^

### Hepatocytes

Hepatocytes comprise the bulk of liver tissue and have a broad range of functions including the metabolism of carbohydrates, fats, and proteins, detoxification of xenobiotic substances, and bile production.

**FIGURE 2:**
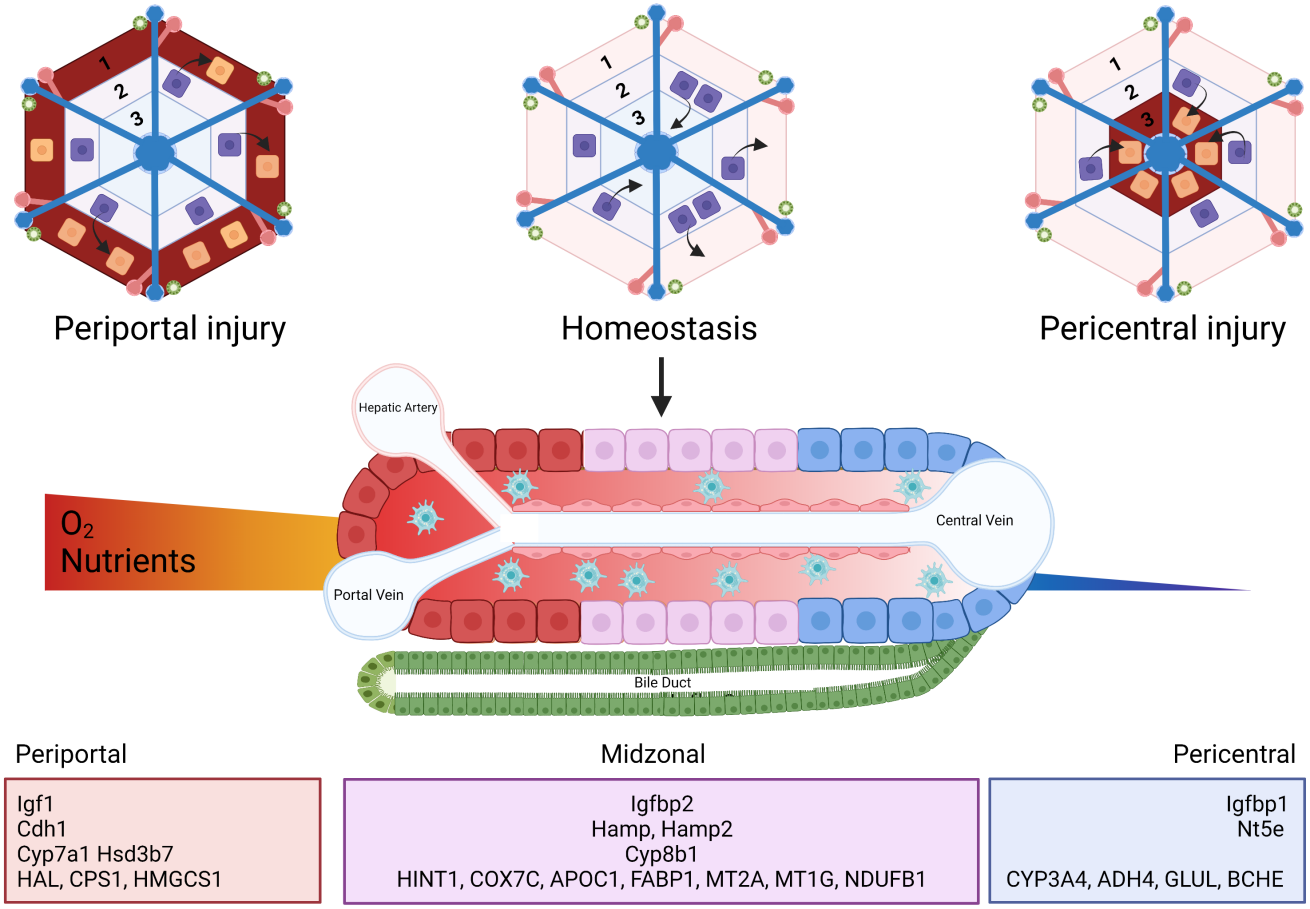
Hepatocyte zonation during homeostasis and regeneration. Through novel and distinct but complementary lineage tracing models, all hepatocytes have been shown to possess proliferative potential,^[^ ^35^, ^40^, ^41^, ^43^, ^44^ ^]^ with midzonal hepatocytes primarily responsible for proliferation during homeostasis,^[^ ^43^, ^44^ ^]^ compared with regional or diffuse hepatocyte proliferative responses following injury. In homeostasis, hepatocyte gene expression varies across the lobule,^[^ ^26^, ^27^, ^28^, ^29^, ^30^ ^]^ with distinct expression profiles for pericentral, midzonal, and periportal hepatocytes. Created with BioRender.com

A seminal study by Halpern et al. combined scRNA‐Seq with smFISH to interrogate hepatocyte zonation within the murine liver.^[^ ^26^ ^]^ Recognizing that whole‐genome reconstruction of liver zonation would require a technique to simultaneously measure the entire transcriptome and the lobule coordinates of many liver cells, the authors selected a panel of six landmark genes on the basis of high expression and diverse zonation patterns. They used these genes to generate a nine‐layer zonation profile of the hepatic lobule, which was subsequently used to infer the location of 1,500 dissociated liver cells, sequenced by MARS‐Seq. Having validated their technique with smFISH on an additional 20 genes, Halpern et al. went on to show that 50% of hepatocyte genes are zonated (Figure 2) and that zonated gene expression is influenced by Wnt, hypoxia, Ras, and pituitary hormones. Kyoto Encyclopedia of Genes and Genomes (KEGG) pathway analysis confirmed previous understanding of zonated pathways, including oxidative phosphorylation and secreted proteins in the periportal zone compared with xenobiotic metabolism, glutathione metabolism, bile acid biosynthesis, and proteasome components in the pericentral zone. In addition, the authors identified genes with nonmonotonic expression, peaking in the midlobule, including hepcidin antimicrobial peptide (*Hamp)* and *Hamp2*, which encode hepcidin, an iron regulating liver hormone. The study also identified pathway genes acting sequentially in zonally adjacent cells. For example, they reported *Igf1* expression periportally, *Igfbp2* expression in the midzone, and *Igfbp1* expression pericentrally, possibly consistent with a negative feedback mechanism, given that Igfbps bind Igf1 and prevent receptor interactions. Expression of zonally adjacent genes was also identified for bile acid synthesis, with expression of *Cyp7a1* and *Hsd3b7* pericentrally and *Cyp8b1* in the midzone.

In a follow‐up study from the same group, Ben‐Moshe et al. extended the initial scRNA‐Seq work to examine both microRNA and protein in the mouse liver.^[^ ^27^ ^]^ Capitalizing on the zonated markers identified previously,^[^ ^26^ ^]^ they FACS sorted cells according to their position within the liver lobule, naming the method “spatial sorting.” Using CD73 encoded by *Nt5e* as a pericentral marker and E‐cadherin encoded by *Cdh1* as a periportal maker, they sorted hepatocytes into eight gates based on graded expression of CD73 and E‐cadherin. Bulk RNA‐Seq of 10,000 sorted cells from each gate yielded results consistent with the previous spatially resolved scRNA‐Seq map, thereby validating the methodology. They then carried out mass spectrometry of 100,000 sorted cells from each zone, demonstrating an overall correlation between mRNA and protein levels, with some exceptions, particularly for secreted proteins such as albumin. Overall, 55% of hepatocyte proteins were zonated, and zonation was correlated at mRNA and protein levels, with notable exceptions; for example, Hnf4α was zonated only at the protein level, with a periportal bias. Also zonated at protein level but not at mRNA level were Rbp4, Idh3b, and Mrpl43, whereas A1cf, Clmn, and Lsr were zonated at mRNA level but not protein level. MacParland et al. published the first human liver scRNA‐Seq data set, sequencing a total of 8,444 cells from the livers of five donors who were neurologically deceased.^[^ ^28^ ^]^ They identified 20 hepatic cell populations, including six hepatocyte clusters. By comparing gene expression in the human hepatocyte clusters with zonated gene expression reported in the mouse,^[^ ^26^ ^]^ they reported significant correlation for four of six clusters, representing periportal, pericentral, and interzonal layers. Pathway analysis identified cholesterol and sterol synthesis and immune processes periportally, contrasting with drug metabolism, Wnt activation, hypoxia, glycolysis, and amino acid biosynthesis pericentrally.

Aizarani et al. then published a healthy human liver atlas consisting of 10,372 cells from nine donors.^[^ ^29^ ^]^ Reasoning that the major axis for variability in any cell type would reflect gene expression changes associated with zonation, they ordered hepatocytes and liver sinusoidal endothelial cells (LSECs) by diffusion pseudotime. In this way, they showed zonation of 41% of hepatocyte genes. Pathway enrichment analysis showed periportal enrichment of genes involved in biological oxidation and glycogen synthesis, whereas midzonal hepatocytes showed enrichment of cytochrome P450 xenobiotic metabolism genes. Notably, comparison of mouse and human liver zonation gene expression showed only limited evolutionary conservation.

Recently, Andrews et al. reported the results of scRNA‐Seq and snRNA‐Seq on liver cells from four human donors.^[^ ^30^ ^]^ By scaling and normalizing individually and then integrating using an analysis approach called Harmony, the authors were able to cocluster the scRNA‐Seq and snRNA‐Seq data sets. They showed that the two sequencing methods favor differing cell types; scRNA‐Seq captured a higher proportion of immune cells whereas snRNA‐Seq captured 50% more cholangiocytes and mesenchymal cells. Six hepatocyte clusters were identified, and correlation with zonated gene expression from the mouse^[^ ^26^ ^]^ identified three of these as either pericentral (*CYP3A4*, *ADH4*, *GLUL*, *BCHE*) or periportal (*HAL*, *CPS1*, *HMGCS1*), with marker genes validated by immunohistochemistry. Two hepatocyte clusters were proposed to correlate with mouse interzonal layers, and validation of the human cluster markers (*HINT1*, *COX7C*, *APOC1*, *FABP1*, *MT2A*, *MT1G*, *NDUFB1*) with immunohistochemistry and spatial transcriptomics confirmed a lack of periportal or pericentral marker expression. The authors stated that the remaining hepatocyte cluster requires further characterization. Interestingly, the authors found that although both snRNA‐Seq and scRNA‐Seq captured hepatocytes equally well, snRNA‐Seq favored pericentral hepatocytes, and scRNA‐Seq favored interzonal hepatocytes. Furthermore, snRNA‐Seq appeared to capture hepatocytes with superior viability, judging by a broad increase in expression of hepatocyte‐related pathways compared with scRNA‐Seq.

Spatial mass spectrometry has also been applied to mouse and human liver to investigate zonation in health and disease.^[^ ^31^ ^]^ Hall et al. used a combination of bulk RNA‐Seq, liquid chromatography‐mass spectrometry, and matrix‐assisted laser desorption ionization‐mass spectrometry imaging to characterize hepatic changes in dietary and genetic mouse models of NAFLD and NASH as well as in human liver biopsies from NAFLD patients with varying degrees of steatosis and fibrosis. The authors reported similar lipid zonation patterns in healthy human and mouse liver, including phosphatidylcholine (32:0) periportally. Progression to NASH was associated with a loss of lipid zonation, which the authors proposed may reflect changes in the zonal location of LPCAT2, a remodeling enzyme that incorporates arachidonic acid into the phospholipid cell membrane, where it serves as a substrate for proinflammatory eicosanoid production.^[^ ^31^ ^]^

The relationship between liver cell ploidy and zonation has also been investigated. In contrast to most tissues, hepatocytes may contain one or two nuclei, each with two, four, or more haploid chromosome sets. In mice, hepatocytes are diploid at birth, and then become increasingly polyploid from weaning onwards.^[^ ^32^ ^]^ Tanami et al. used single molecule‐based tissue imaging to examine the distribution of hepatocyte polyploid classes in mice of different ages. They reported that hepatocyte polyploidy advances more rapidly in the midlobule zone compared with the periportal and pericentral zones.^[^ ^33^ ^]^ To investigate crosstalk between ploidy and zonation in regulation of gene expression, Richter et al. FACS sorted 2n and 4n hepatocytes based on their genome content and carried out snRNA‐Seq.^[^ ^34^ ^]^ They showed that the median number of genes detected in 4n hepatocytes was 1.25‐fold higher than in 2n hepatocytes. Furthermore, despite a similar overall transcriptional profile, 312 differentially expressed genes were identified between the two cellular states. Using diffusion pseudotime to infer hepatocyte position within the lobule, they showed a 1.3‐fold enrichment for 4n hepatocytes in the pericentral cluster, consistent with previous findings.^[^ ^33^, ^35^ ^]^ Although 55 of the 224 zonation markers up‐regulated in the pericentral cluster were up‐regulated only in 4n hepatocytes, it was clear that hepatic metabolic zonation was present in both 2n and 4n hepatocytes, irrespective of ploidy status. The authors concluded that a combination of ploidy and position within the liver lobule determines hepatocyte gene expression.

## ZONATION OF LIVER REGENERATION

The liver’s remarkable capacity to regenerate following injury has been known for thousands of years; however, the key cell types involved and their location within the liver continue to be a very active area of research.

Wang et al. proposed pericentral Axin2^+^ hepatocytes as a source of new hepatocytes following liver injury.^[^ ^36^ ^]^ They used a tamoxifen‐inducible Axin2‐CreERT2;Rosa26‐mTmG^flox^ mouse to pulse label Axin2^+^ cells and showed an expansion of labeled cells over time, spreading from the central vein toward the portal vein and accounting for 40% of hepatocytes within one year.

Around the same time, two other groups proposed a periportal hepatocyte population that mediates liver repair following injury. Font‐Burgada et al. reported that a population of Sox9^+^ periportal hepatocytes proliferate extensively and replace liver mass after chronic hepatocyte injury,^[^ ^37^ ^]^ whereas Pu et al. reported that Mfsd2a^+^ hepatocytes located in the periportal zone repair the liver following partial hepatectomy and also following chronic CCl_4_‐induced liver injury.^[^ ^38^ ^]^ In contrast, Lin et al. used lineage tracing from the telomerase reverse transcriptase (Tert) locus in mice to show that rare hepatocytes with high telomerase expression are distributed throughout the liver lobule and are responsible for hepatocyte regeneration and repair in homeostasis and following injury respectively.^[^ ^39^ ^]^

In 2020, a trio of papers^[^ ^35^, ^40^, ^41^ ^]^ using varied but complementary approaches demonstrated that hepatocytes, irrespective of position within the liver lobule, possess regenerative capacity. In the 2015 study by Wang et al., insertion of a CreERT2 cassette into the endogenous Axin2 locus also disrupted one *Axin2* allele, a protein essential for β‐catenin degradation.^[^ ^36^ ^]^ Recognizing this as a potential confounding factor, Sun et al. used bacterial artificial chromosome (BAC)‐transgenic Axin2CreERT2 mice bred to R26‐EGFP reporter mice to label β‐catenin active cells without affecting function.^[^ ^40^ ^]^ This model accurately reported β‐catenin activation in Zone 3, but positive cells did not spread to other zones, even up to 10 months after trace initiation. Following ablation of Axin2^+^ Zone 3 cells by diphtheria toxin expression, damage was repaired by remaining Zone 3 and adjacent Zone 2 cells. Similarly, partial hepatectomy resulted in regeneration by hepatocytes from all liver zones, as demonstrated by EdU incorporation. Sun et al.’s findings were further supported by a lineage tracing study using the BAC Lgr5^+^ reporter mouse, in which labeled Zone 3 cells failed to contribute to hepatocytes in other zones.^[^ ^42^ ^]^

Further studies also corroborated the conclusion that hepatocytes, irrespective of zonal position, possess regenerative capacity.^[^ ^35^, ^41^ ^]^ Chen et al., through AAV8‐TBG‐Cre injection into adult heterozygous Rosa26‐Rainbow Cre reporter mice, labeled random hepatocytes with one of three fluorophores at a neutral genetic locus.^[^ ^41^ ^]^ Multiparameter imaging showed an absence of large clusters or multicellular clones up to 17 months after tracing initiation. Modeling acute and chronic injury with CCl_4_ and allyl alcohol for pericentral and periportal insults, respectively, resulted in repair of injury by adjacent hepatocytes.

Matsumoto et al. used Rosa‐Confetti multicolor reporter mice bred to Ubc‐CreERT2 transgenic mice to label hepatocytes with one or more colors following tamoxifen injection, depending on ploidy status. Using CCl_4_, 3,5‐diethoxycarbonyl‐1,4‐dihydrocollidine diet, and the fumarylacetoacetate hydrolase (FAH) model to induce liver injury, they showed that clonal proliferation of labeled hepatocytes was not restricted to a specific subset of cells but was instead dictated by the location of injury.^[^ ^35^ ^]^

Most recently, two studies have suggested that liver homeostasis is preferentially maintained by Zone 2 hepatocytes . He at al. developed a novel lineage tracing model (ProTracer) that capitalizes on the use of two orthogonal site specific recombinases, Cre and Dre, to continuously label Ki67^+^ proliferating cells.^[^ ^43^ ^]^ They showed that midzonal hepatocytes are primarily responsible for maintaining hepatocyte mass during homeostasis, with less proliferation in periportal hepatocytes and minimal proliferation in pericentral hepatocytes. In agreement with these findings, Wei et al. used a panel of 11 novel *CreER* knock‐in mouse lines to label zonal subpopulations across the liver lobule.^[^ ^44^ ^]^ They showed that over time under homeostatic conditions, Zone 1 and Zone 3 hepatocytes decreased in number. In contrast, midlobular Zone 2 hepatocytes marked by the *Hamp2* gene were responsible for the majority of regeneration. To investigate the pathways regulating Zone 2 proliferation, bulk and scRNA‐Seq were used to analyze differentially expressed genes, ultimately identifying the IGFBP2‐mTOR‐CCND1 axis as one mechanism responsible for preferential repopulation of Zone 2 hepatocytes during homeostasis. In spite of the midzonal regenerative bias reported by He et al. and Wei et al. in homeostasis, repopulation following injury was highly regional and dependent on the location of liver injury,^[^ ^43^, ^44^ ^]^ consistent with the previous studies described above (Figure 2).

### Cholangiocytes

Cholangiocytes are the epithelial cells that line bile ducts. They comprise 3%–5% of total human liver cells and produce 40% of bile volume. Because cholangiocytes comprise a small percentage of total liver cells and have historically proven hard to purify, cholangiocyte data provided by scRNA‐Seq studies have been limited.^[^ ^28^ ^]^ However, the introduction of snRNA‐Seq has enabled interrogation of cholangiocyte zonation within the liver for the first time.^[^ ^30^, ^45^ ^]^

Andrews et al. demonstrated that snRNA‐Seq captured more cholangiocytes than scRNA‐Seq from healthy human liver, generating a combined data set of 448 *ANXA4*, *SOX9*, and *KRT7* expressing cholangiocytes. Subclustering revealed six transcriptionally distinct populations, including three hepatocyte‐like *ASGR1* ^+^ clusters, two cholangiocyte‐like clusters (marked by *KRT7*, *KRT18*, *SLC4A2*), and one progenitor cluster. The clusters formed a branching trajectory from bipotent progenitors toward differentiated hepatocytes and cholangiocytes. Two cholangiocyte populations captured only by snRNA‐Seq were interrogated spatially, using immunohistochemistry and spatial transcriptomics. Specifically, a less differentiated cholangiocyte population, determined to be small cholangiocytes on the basis of *BCL2* expression and bile duct restricted staining, and a bipotent progenitor population positive for *FOXO3*, *FGFR3*, *ANPEP*, and *GATA6* marker genes were interrogated.

### Liver endothelial cells

The liver vascular endothelium is comprised of LSECs as well as the endothelium of blood vessels. Before the recent flurry of scRNA‐Seq studies, immunostaining studies had already demonstrated zonation of LSECs in the liver, with LYVE1 and CD14 identified as markers that distinguish midzonal and central LSECs from periportal LSECs.^[^ ^46^, ^47^, ^48^ ^]^

**FIGURE 3:**
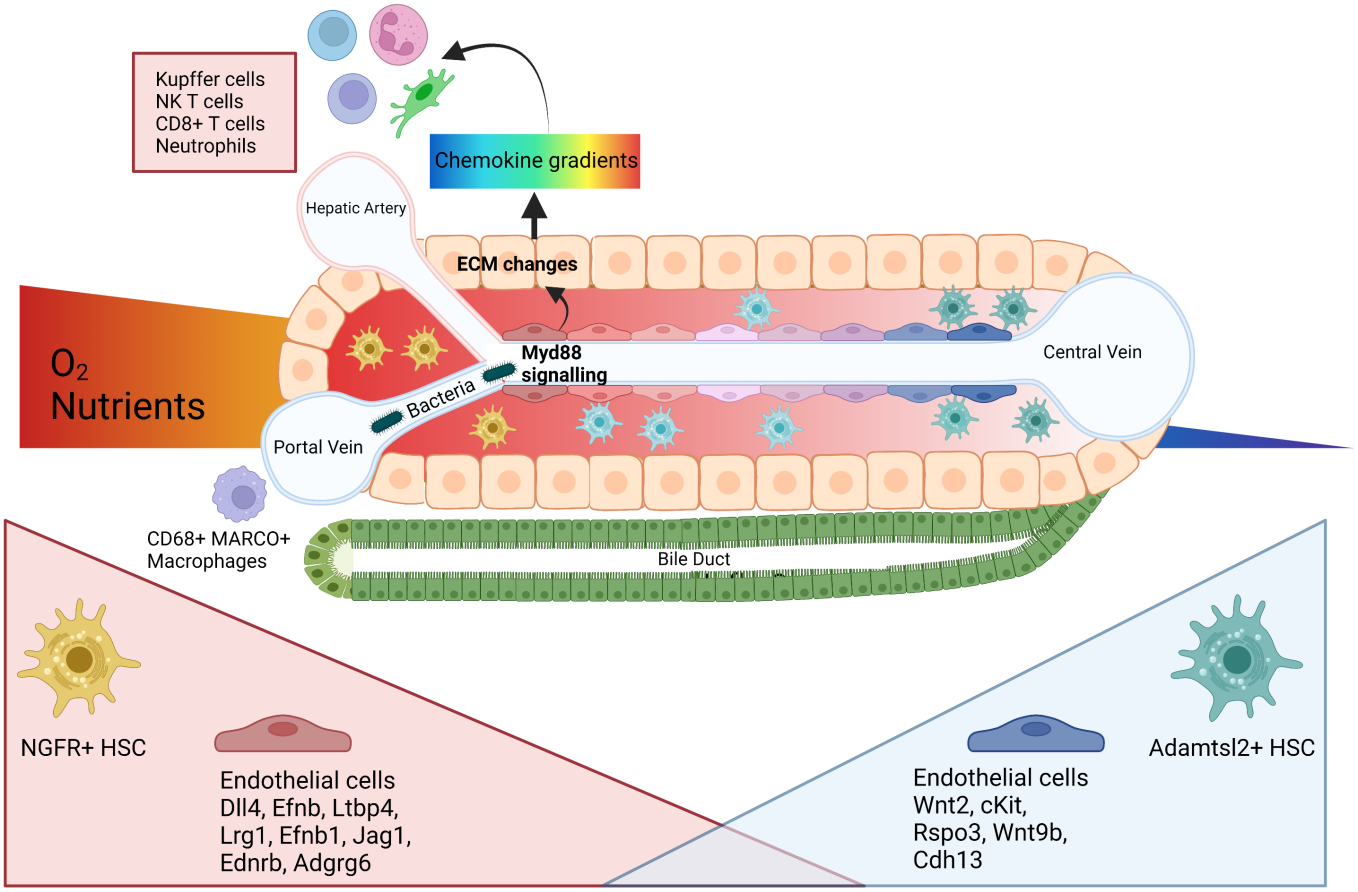
Hepatic nonparenchymal cell zonation. Immune zonation is driven by gut‐derived microbiota in the portal circulation, activating Myd88 signaling in LSECs and modulating ECM characteristics and therefore chemokine gradients.^[^ ^59^ ^]^ Periportal accumulation of Kupffer cells, NK T cells, neutrophils, and CD8+ T cells is observed.^[^ ^59^ ^]^ Tolerogenic CD68+ Marco+ macrophages occupy the periportal niche.^[^ ^28^ ^]^ Variable gene expression is also observed in endothelial cells and HSCs across the lobule. Periportal HSCs are NGFR+ and pericentral HSCs are Adamstl2+,^[^ ^54^ ^]^ whereas endothelial cells exhibit a spectrum of transcriptomic profiles across the hepatic lobule^[^ ^49^ ^]^. Created with BioRender.com

Zonation of liver endothelial cells (Figure 3) was first examined using scRNA‐Seq in mouse liver.^[^ ^49^ ^]^ Given the small size of endothelial cells and the scarcity of established endothelial landmark genes, Halpern et al. developed a novel method termed “paired cell sequencing” in which they sequenced pairs of hepatocytes and endothelial cells and then consulted their previously established hepatocyte zonation profile^[^ ^26^ ^]^ to infer endothelial location within the lobule. Significant zonation was identified for 35% of the 1,303 genes specifically expressed in endothelial cells, and the findings were validated by smFISH for 12 of these: pericentral *Rspo3*, *Cdh13*, *Thbd*, *Wnt2*, and *Kit*; periportal *Dll4*, *Efnb2*, and *Ltpp4*; and nonmonotonic *Lyve1, Ccnd1*, *Bmp2*, and *Stab1*. The authors subsequently developed a panel of endothelial landmark genes (70 pericentral, 70 periportal) to create an endothelial liver zonation map, facilitating the study of zonation patterns in genes expressed by hepatocytes and endothelial cells alike. Finally, they identified c‐Kit surface expression on endothelial cells as a marker which decreases along the central to portal axis. Zonation of liver endothelial cells has also been demonstrated in the healthy human liver.^[^ ^29^ ^]^ In this study, 67% of LSEC genes were zonated, with central and midzonal endothelial cells exhibiting peak expression of *LYVE1* and *FCN3*.

Liver endothelial zonation has also been examined in the context of disease. Xiong et al. performed scRNA‐Seq on liver cells from mice fed either chow diet or Amylin diet (an established model of NASH) for 20 weeks.^[^ ^50^ ^]^ Four endothelial clusters were identified and labeled as pericentral or periportal endothelial cells and LSEC 1 and LSEC 2. Correlating the data with zonated endothelial genes previously validated by smFISH,^[^ ^49^ ^]^ gene expression for the clusters was described, including pericentral endothelial: *Wnt9b*, *Rspo3*, *Cdh13*, and *Wnt2*; and periportal endothelial: *Ednrb*, *Jag1*, *Lrg1*, *Efnb1*, *Ltbp4*, and *Adgrg6*. LSEC populations were characterized by high expression of *Fcgr2b* and *Gpr182*, known LSEC markers. The authors reported NASH‐induced alterations of gene expression that appeared to occur in all four endothelial clusters equally. Changes in NASH included increased LSEC expression of *Cxcl9* and *BODIPY* and a reduced abundance and altered histological integrity of the liver sinusoids.

Liver endothelial zonation was investigated in cirrhosis by Su et al., who carried out scRNA‐Seq on endothelial cells harvested from the liver of endothelial GFP reporter mice subjected to 12 weeks of CCl_4_ inhalation, and healthy controls.^[^ ^51^ ^]^ Su et al. identified six clusters of endothelial cells, including three LSEC clusters that they assigned to Zones 1–3 of the liver lobule based on previously published landmark endothelial genes.^[^ ^49^ ^]^ The authors observed significant changes in LSEC gene expression that were most marked in Zone 3. These included up‐regulation of genes consistent with capillarization (*CD34*) and ECM (*Col4a1*, *Col4a2*, *Col5a2*, and *Fbn1*) and decreased expression of endocytic receptors including manose receptor (*Mrc1*) and scavenger receptors (*Stab1*, *Stab2*, *Scarb1*, and *Scarb2*) as well as lysosomal transport protein (*Lamp2*). Transcription factors (*Klf2* and *Klf4* and *AP‐1*) that mediate nitric oxide production in response to shear stress were down‐regulated in LSECs across all zones in cirrhotic mice, implying increased intrahepatic vascular resistance in the setting of fibrosis.

Scar‐associated endothelial subpopulations have also been identified in human liver.^[^ ^52^ ^]^ Ramachandran et al. compared scRNA‐Seq data from healthy human liver tissue and human liver tissue with cirrhosis, identifying distinct populations of disease‐associated CD34+PLVAP+VWA1+ and CD34+PLVAP+ACKR1+ endothelial cells. These cells were not present in healthy livers and were spatially restricted to the fibrotic niche of diseased livers. The VWA1+ cluster expressed profibrogenic genes including *PDGFD*, *PDGFB*, *LOX*, and *LOXL2*, whereas the population marked by *ACKR1* displayed an immunomodulatory phenotype. Functionally, disease‐associated endothelial cells displayed enhanced leukocyte transmigration, suggesting a role in the regulation of hepatic inflammation.

## MESENCHYMAL CELLS

Mesenchymal cells within the liver comprise hepatic stellate cells (HSCs), fibroblasts (FBs), and vascular smooth muscle cells (VSMCs). HSCs are found in the subendothelial space of the liver sinusoid, known as the space of Disse, and are the main storage site for vitamin A. Intralobular heterogeneity of HSCs was described in 1993 in the pig^[^ ^53^ ^]^, however, recent application of scRNA‐Seq in this context has facilitated high‐resolution interrogation of HSC heterogeneity.

Dobie et al. performed scRNA‐Seq on over 30,000 mouse hepatic mesenchymal cells isolated from PDGFRb‐GFP knock‐in mice, identifying three main mesenchymal populations that they validated using immunofluorescence staining.^[^ ^54^ ^]^ They described CD34^+^ FBs located primarily in the portal niche, Reelin^+^ HSCs within the perisinusoidal space throughout the liver, and calponin 1^+^ VSMCs within the hepatic artery and portal vein walls. Equivalent populations were demonstrated in human liver using MFAP4 (FBs), RGS5 (HSCs), and MYH11 (VSMCs) as markers. Further bioinformatics analysis of the healthy HSCs suggested two HSC subpopulations. Using an independent component analysis approach, they generated markers for these two HSC subpopulations, and immunofluorescence staining plus smFISH demonstrated that HSCs partition into topographically diametric lobule regions, designated portal vein‐associated HSCs (PaHSCs) and central vein‐associated HSCs (CaHSCs) (Figure 3). PaHSCs are marked by NGFR expression, and CaHSCs are marked by AdamtsI2 expression. These zonated HSC genes were conserved following centrilobular (CCl_4_‐induced) liver injury. Finally, the authors created a myofibroblast signature by thresholding HSCs on fibrillar collagen expression and mapped this onto the zonation profile. This approach identified CaHSCs as the predominant collagen producing HSC in acute and chronic CCl_4_‐induced liver injury, demonstrating spatial and functional zonation of HSCs. An additional finding of the study was that *Itgb3* and *Rspo3*, previously used as landmark genes to zonally define endothelial cells,^[^ ^49^ ^]^ also zonally defined HSC, suggesting a degree of commonality of zonal marker genes between these two cell lineages.

Heterogeneity of HSCs in healthy and injured liver was also demonstrated in a study by Rosenthal et al.^[^ ^55^ ^]^ Here, scRNA‐Seq was used to evaluate HSCs from *foz/foz* mice fed a chow diet (control) or a Western diet (NASH model) for 12 weeks and from the same NASH mice 8 weeks after being switched back to a chow diet, i.e., in fibrosis regression. They showed two clusters of quiescent HSCs in control liver, correlating with periportal and pericentral populations based on significant overlap with previously reported gene expression (33/46 and 20/26 genes, respectively).^[^ ^54^ ^]^ In NASH, four populations of mesenchymal cells were identified, described as classic myofibroblast (*Col1A1*, *Timp2*, and *Acta2*), intermediate activated (lower expression of fibrogenic genes, but expression of markers including *IRF7*), a novel immune/inflammatory cluster (*Cd36*, *Ly6c*, and *CLEC*), and proliferating (*Cdk1*). Finally, an inactivated population was detected in regression, with unique markers including *Cxcl1*, *Gabra3*, and *Fbln7*, and reappearance of quiescence related genes including *Bambi*, *Vipr1*, and *ApoE*. Interestingly, they showed that both clusters of quiescent HSCs contributed equally to activated HSC phenotypes, further demonstrating that the zonal HSC population activated likely reflects the location of injury, for example, centrilobular injury in CCl_4_ and diffuse metabolic dysregulation‐mediated injury in NASH.

Zonation of human liver mesenchymal cells has also been reported. Akin to their findings in the mouse, Dobie et al. observed zonated expression of *NGFR* and *ADAMTSL2* across the lobule of healthy human liver.^[^ ^54^ ^]^ Andrews et al. subsequently used a combination of scRNA‐Seq and snRNA‐Seq to interrogate mesenchymal cells in healthy human liver, showing improved capture of mesenchymal cells using snRNA‐Seq compared with scRNA‐Seq (2.5% vs. 1%, respectively).^[^ ^30^ ^]^ Integration of the data sets identified seven mesenchymal clusters, including three HSC clusters, two FB clusters, and two VSMC clusters based on cross‐species correlation analysis of healthy and diseased mouse hepatic mesenchyme gene expression data.^[^ ^54^ ^]^ Spatial transcriptomics showed that HSC gene expression was dispersed throughout the liver lobule, whereas FBs and VSMCs were predominantly located periportally, as shown previously. Mesenchymal cell zonation in human liver disease warrants further investigation, and this is an ongoing area of research in the field.

## IMMUNE CELLS

Immune zonation within the liver (Figure 3) is an evolving area of research . scRNA‐Seq of healthy human liver immune cells reported two populations of CD68^+^ macrophages, categorized as inflammatory (*MARCO* negative) and tolerogenic (*MARCO* positive) phenotypes.^[^ ^28^ ^]^ IHC staining showed a preponderance of the tolerogenic MARCO positive population in the periportal area, suggestive of functional zonation of macrophage subsets.

Similar findings were reported in a combined scRNA‐Seq, snRNA‐Seq, and spatial transcriptomics study of healthy human liver, in which spatial transcriptomics demonstrated that the noninflammatory macrophage genes and gene signature were present in the periportal regions, whereas the inflammatory macrophage genes and gene signature were expressed around the central vein.^[^ ^30^ ^]^

Enrichment of Kupffer cells with specific properties in the periportal regions of mouse liver has been recognized for many years.^[^ ^56^, ^57^, ^58^ ^]^ However, recent work in the mouse has provided novel insights regarding the spatial organization of liver immune cells and the mechanisms responsible.^[^ ^59^ ^]^ Gola et al. used a combination of imaging techniques (multiparameter confocal imaging, intravital two‐photon microscopy, clearing‐enhanced three‐dimensional imaging) to demonstrate that Kupffer cells and natural killer (NK) T cells are preferentially located in periportal regions. This immune zonation was not altered by disrupted Wnt–β‐catenin signaling in hepatocytes but was instead shown to be a dynamic process initiated at weaning and dependent on gut microbiota. Furthermore, the authors showed that commensal bacteria sustain Myd88 signaling in LSECs and that this determines extracellular matrix (ECM) characteristics and therefore chemokine gradients, which dictate lymphoid and myeloid cell immune zonation in the liver. Using *Listeria monocytogenes* bacteria and malaria sporozoite challenge models, the authors showed that neutrophils and CD8^+^ T cells also exhibit periportal immune zonation. Critically, disruption of Myd88, Cxcr3, or Cxcl9 chemokine signaling decreased liver capture of *L. monocytogenes* and potentiated systemic bacterial dissemination, demonstrating the importance of immune zonation in the liver.

## FUTURE PERSPECTIVES

Recent technological advances have driven an exponential increase in understanding of liver zonation, and this area of research continues apace. For example, with the introduction of snRNA‐Seq, liver zonation can now also be examined across a range of human liver diseases, using the arrays of frozen, biobanked human liver tissue archived in repositories around the world. Furthermore, development of innovative methodologies in this space will undoubtedly hasten discoveries, for example, “paired sequencing” for understanding of liver zonation in scarce cell types^[^ ^49^ ^]^ and spatial sorting^[^ ^27^ ^]^ to enable collection of sufficient zonated cellular material for further analysis.

Sun et al. recently used a combination of ATAC‐Seq and RNA‐Seq to interrogate the differential outcomes of Wnt signaling in Axin2^+^ hepatocytes compared with Axin2^+^ intestinal stem cells.^[^ ^60^ ^]^ They showed that although proliferation‐related β‐catenin regulated genes were equally accessible in both cases, the balance of ZNRF3 and RNF43 in the liver was critical to fine‐tune Wnt signaling, ultimately limiting hepatocyte proliferation and maintaining metabolic zonation. The use of single‐cell ATAC‐Seq has been used to study functional diversification of hepatocytes during liver regeneration,^[^ ^61^ ^]^ and this approach will undoubtedly be used in the future to further elucidate hepatocyte heterogeneity in the context of liver zonation.

Development of spatial transcriptomic^[^ ^68^ ^]^ and spatial proteomic technologies will vastly enhance future study of liver zonation. In a recent study, Guilliams et al. produced a spatial proteogenomic atlas of the healthy human and murine liver by combining cellular indexing of transcriptomes and epitomes by sequencing (CITE‐seq), sc‐RNASeq, sn‐RNASeq, Molecular Cartography (Resolve BioSciences) for 100‐plex spatial mRNA analysis, spatial transcriptomics, and spatial proteomics.^[^ ^45^ ^]^ The authors aligned this atlas across seven species, thus establishing the location of hepatic Kupffer cells and identifying Gpnmb‐expressing bile duct macrophages, consistent with previously reported lipid‐associated macrophages (LAMs).^[^ ^62^ ^]^

Future work will also delve deeper into the molecular mechanisms regulating zonation, not only increasing our understanding of how liver zonation occurs but also generating tractable targets that may allow therapeutic manipulation of zonation in the context of disease. For example, Gola et al. demonstrated that immune zonation within the liver is a dynamic and reversible process, dependent on the continued presence of gut microbiota, and disrupted by the administration of antibiotics.^[^ ^59^ ^]^ Given that liver zonation is known to be essential for normal metabolic function within the liver,^[^ ^16^, ^17^ ^]^ zonation‐preserving therapies, perhaps through manipulation of the gut microbiota, may uncover unexplored therapeutic avenues in patients with liver disease. Further therapeutic strategies for targeting liver zonated genes may include the use of adeno‐associated viral vectors,^[^ ^63^ ^]^ antisense oligonucleotides,^[^ ^64^ ^]^ and genome editing, for example, by CRISPR technologies.^[^ ^65^ ^]^

Recognizing that studies of liver zonation also need to account for circadian rhythms, in particular the role of fasting and sleep cycles, Droin et al. recently reported results of scRNA‐Seq of whole mouse liver obtained at four equally spaced time points in a 24 h period.^[^ ^66^ ^]^ Using mixed‐effect models of mRNA expression and validation with smFISH, they showed that 7% of genes expressed within the liver were both zonated and rhythmic. Unsurprisingly, core circadian clock genes were expressed in a nonzonated manner, showing that the liver clock remains independent to zonal influences. KEGG pathway analysis revealed previously unreported zonated pathways, for example accumulation of cytosolic chaperones centrally and endoplasmic reticulum chaperones portally, with both peaking during the activity/feeding period, probably reflecting an increased need for protein folding during this phase. Mechanistically, they found that Wnt activity was rhythmic within the liver, identifying functionally important Wnt targets that were strongly rhythmic in expression at the protein level, including CYP7A1, FMO5, coumarin 7‐hydroxylase, Slc1a2, and ABCC2. These findings suggest that future attempts to therapeutically manipulate liver zonation patterns in the context of disease will also need to take into account the underlying circadian rhythms of zonation within the human liver.

Finally, the concept of zonation plasticity in the liver merits further investigation. Studies of liver repair following injury have shown changes in gene expression as liver cells move from one zone to another to align with local zonation patterns,^[^ ^38^, ^40^ ^]^ as might be expected in light of previous work on morphogen gradients (such as Wnt) within the liver.^[^ ^17^ ^]^ Given that cellular gene expression, and hence cellular phenotype, alters in a zone‐dependent manner in the context of disease,^[^ ^31^, ^51^ ^]^ therapies geared toward “zonal‐switching” of cell state and phenotype may open up a broad range of novel precision medicine‐based approaches to treat patients with liver disease.

**TABLE 1:** Current understanding of mechanisms regulating liver zonation by cell type

Cell type	Regulator of zonation	References
Hepatocyte	Oxygen	^[^ 15, 26 ^]^
	Wnt/β‐catenin	^[^ 14, 16, 17, 18, 26, 27, 60, 66 ^]^
	RSPO family	^[^ 20, 21 ^]^
	Ras signaling	^[^ 18, 19, 26 ^]^
	DICER	^[^ ^22^ ^]^
	HNF4α	^[^ ^23^ ^]^
	ZNRF3	^[^ ^60^ ^]^
	RNF43	^[^ ^60^ ^]^
	Pituitary hormones	^[^ ^26^ ^]^
	IGFBP2‐mTOR‐CCND1 axis	^[^ ^44^ ^]^
Immune cells	Gut microbiota/Myd88 LSEC signaling	^[^ ^59^ ^]^
	Cxcr3	^[^ ^59^ ^]^
	Cxcl9	^[^ ^59^ ^]^

## CONFLICTS OF INTEREST

Nothing to report.

## AUTHOR CONTRIBUTIONS

Jasmin Paris and Neil C. Henderson contributed equally to the writing and editing of all aspects of this review.
